# High protein intake during the early phase of critical illness: yes or no?

**DOI:** 10.1186/s13054-018-2196-5

**Published:** 2018-10-25

**Authors:** Jean-Charles Preiser

**Affiliations:** Department of Intensive Care, Erasme University Hospital, Université Libre de Bruxelles, 808 route de Lennik, B-1070 Brussels, Belgium

**Keywords:** Nitrogen, Muscle weakness, Anabolic resistance, Insulin resistance, Amino acids, Medical nutrition, Enteral, Parenteral

## Abstract

The rationale for the provision of nitrogen from proteins given via the enteral route or from intravenous amino acids is to boost the synthesis of muscle proteins, and thereby to limit the severity of intensive care unit-acquired weakness by the prevention of muscle loss. However, the optimal timing for supplemental nitrogen provision is a matter of debate and controversy. Indeed, consistent data from retrospective studies support an association between high early protein intakes and better outcomes, while recent post-hoc findings from prospective studies raise safety concerns. This pro–con paper details the arguments of both sides and highlights the need for large-scale prospective studies assessing the safety and efficacy of different levels of protein intake in combination with physical activity and summarizes the currently recruiting clinical trials.

## Introduction

A large consensus supports the provision of high protein intakes during the late phase of critical illness, e.g., during recovery when the ability to increase the synthesis of muscle proteins from the pool of circulating amino acids increases [1, 2]. However, controversial views are expressed regarding the amount of proteins to be given during the early phase of critical illness, when muscle protein breakdown outweighs muscle synthesis as a result of the resistance to anabolic stimuli [3, 4]. The proportion of nitrogen losses to be compensated by protein intake in the critically ill is a matter of debate, as reflected by recommendations cited in the most recently published guidelines: 1.2–2.5 g/kg of protein per day [5, 6] and the provision of an amount of protein lower than nitrogen losses [1, 4], in agreement with the “Baby stomach” concept [7]. These discrepant views based on experts’ opinions reflect the paucity of data from adequately powered clinical studies assessing the effects of different amounts of proteins on relevant endpoints [8].

Meanwhile, industrial companies recently started to market nutritional formulas containing high amounts of proteins or amino acids and promoted their use early during the course of critical illness, following experts’ opinions mainly based on associations between high nitrogen intakes and better outcomes and on biochemical arguments. The marketing of these solutions is possible as the legal standards do not require the same sequence of testing as for a new drug, i.e., phase I clinical trials to check the safety, phase II clinical trials to assess the efficacy, and phase III clinical trials to compare the new treatment with the current standard of care. In the field of nutrition, this sequence is usually not followed; as a result the issue of safety may have been overlooked [9]. Nonetheless, the issues raised by the three phases of clinical testing are relevant for nutritional solutions as well as for any new therapeutic modality.

**Fig. 1:**
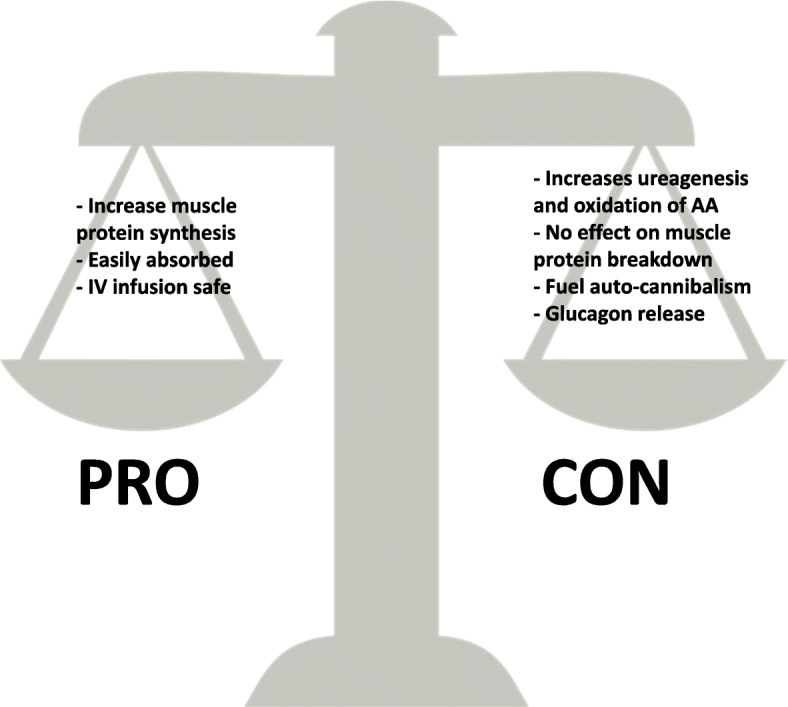
The arguments in favor and against high protein intake during the early phase of critical illness. *AA* amino acid

The community of clinicians is then left with conflicting arguments either supporting the use of high protein solutions or cautioning against this practice (Fig. 1). This manuscript intends to summarize the arguments supporting both sides and the current clinical research.

## High nitrogen intake during the early phase of critical illness: the pros

The renewed enthusiasm for high protein intake results mainly from attention paid to ICU-acquired muscle weakness (ICU-AW). Indeed, the importance of ICU-AW in the outcome of critically ill patients has been underlined by the description of long-term physical impairments and disabilities impairing the quality of life of survivors and increasing healthcare-related costs. The time course of muscle wasting is characterized by an initial abrupt drop in muscle mass and function followed by a slow, progressive recovery [10–13]. Recently, decreased mitochondrial biogenesis and dysregulated lipid oxidation have been reported as contributors to compromised skeletal muscle bioenergetic status [14]. Clinically, in addition to a decrease in functional autonomy and quality of life, this prolonged muscle weakness represents a huge burden for society as a high proportion of patients who required an ICU stay lasting several days are unable to return to work or even to home [15].

The prevention of ICU-AW requires a multi-modal “bundle” approach, including the avoidance of sedation, early mobilization, and ambulation. The inclusion of high protein intakes in this bundle of measures appears logical as an adjunctive measure to limit the loss of muscle mass and function by boosting the synthesis of muscle proteins. High protein intake is expected to stimulate new protein synthesis, thereby preserving muscle mass [6]. The combination of physical activity, including active and passive mobilization, with high protein nutritional formulas or supplemental intravenous amino acids was suggested as a “must” for physical rehabilitation. Research in this area was even ranked as the number one priority by a group of experts [16]. Compelling retrospective data on large cohorts of patients support these expectations, as improved survival was observed in patients who received the highest amounts of protein, regardless of their physical activity [17–20].

The results of a recent clinical prospective study confirm that it is possible to increase the circulating pool of amino acids with an enteral solution containing high amounts of proteins [21], in spite of high splanchnic extraction of some amino acids [22]. In other interventional studies, intravenous infusion of amino acids was found to be safe in patients at risk of acute renal failure [23] and transiently improved muscle function [24]. Improved 90-day survival was even found in a post-hoc analysis in the subset of patients with normal renal function [25].

Parallel to this quantitative approach, the qualitative aspects of proteins can also represent a promising area of clinical research. For instance, whey proteins could increase muscle synthesis more efficiently than soy or casein-based solutions as a result of their higher digestibility, their higher content in leucine, and their insulinotropic properties [26, 27]. Likewise, the effects of semi-elemental or elemental solutions should be re-considered as a means to improve digestibility and protein availability during enteral nutrition [28].

## High nitrogen intake during the early phase of critical illness: the cons

On the “con” side of high protein intake, no clinical benefit has been reported from interventional studies comparing solutions containing high amounts of nutrients, including proteins, with standard amounts [29–32]. However, in contrast to a potential benefit on muscle protein synthesis, the issue of the safety of high nitrogen intake during the acute phase of critical illness is an emerging concern. Indeed, a preplanned post-hoc analysis of the PEPaNIC study [33] that evaluated the effects of withholding parenteral nutrition in critically ill children suggests a linear positive correlation between the amount of amino acids provided and poorer outcome in the children randomized to the early parenteral nutrition group, until day 4 after admission [34] . The underlying mechanisms are not fully understood and are currently being investigated. Besides increased urea generation reported in the EAT-ICU (Early Goal-Directed Nutrition in ICU Patients) trial [35], increased production of glucagon leading to further oxidation of amino acids has also been reported [36].

Teleologically, muscle wasting could be considered a desirable consequence of adaptive anabolic resistance and lasts a few hours or days after injury [37]. The inability to respond to anabolic stimuli during the acute phase can be considered as a component of an adaptive response designed to provide substrates for gluconeogenesis in order to meet the requirements of vital organs and systems, an event known as auto-cannibalization or auto-cannibalism [3]. In this scenario, the loss of muscle would serve to supply gluconeogenetic organs. Likewise, the ability of muscles to build myofibrils will be limited and the provision of high amounts of amino acids will not attenuate the muscle wasting and could even amplify the degradation of amino acids.

## Conclusions

The risk-to-benefit ratio of the provision of high amounts of proteins or amino acids during the early phase of critical illness is largely unknown. Some aspects have been investigated, while others are still unexplored.

Importantly, the optimal combination of proteins and physical activity is unknown [38]. This is a key issue when early physical activity is feasible and probably beneficial. Of note, the needs and protein metabolism of elderly and/or obese patients can differ from those of the overall ICU population [6, 39, 40].

Hopefully, some of the pending issues could be answered by some of the ongoing trials registered on clinicaltrials.gov (Table 1). Most of the currently recruiting studies are prospective randomized controlled trials. The inclusion criteria studies are highly variable, even though an anticipated long stay and the requirement for mechanical ventilation are mandatory in most trials. The primary outcomes tend to focus on physical function in several studies, while all-cause mortality is less commonly used as a primary outcome. A wide range of interventions are being tested and compared to the standards of care, from supplemental proteins (1.5–3.0 g/kg/day) alone to combination with standardized physical activity.

**Table 1:** Ongoing studies currently recruiting adult patients (source: Clinicaltrials.gov – Jul 23, 2018)

NCT number	Design	Region	Inclusion criteria	Primary outcome	Secondary outcomes	Intervention	Comparator	Planned sample size
01833624	Open-label PRCT	France	• Traumatic brain injury • Non-traumatic brain injury: stroke, intracranial and/or subarachnoid hemorrhage, subdural and/or extradural hematoma • Expected duration of mechanical ventilation > 48 h	Nutritional efficacy	Morbidity and mortality	Small-peptide enteral feeding formula	Whole-protein formula	206
02509520	PRCT	USA	• Age ≥ 45 years • Respiratory insufficiency requiring mechanical ventilation • ICU presentation < 6 days • All four limbs intact and mobile • Eligible for and able to participate in physical therapy • Pre-admission Barthel Index > 70	-Muscle mass -Global body strength -Mobility status -Short physical performance battery	-Time to weaning -ICU/hospital length of stay -Discharge disposition -Weaning success	Functional strength and cardiopulmonary endurance training MPR and high protein supplement goal of 1.6 g/kg/day protein	“No intervention”: MPR “Active comparator”: MPR and high protein supplement	60
02106624	PRCT	China	• Need mechanical ventilation for more than 2 days • Mean blood pressure more than 60 mmHg • Predicted ICU stay more than 7 days • Tolerance of parenteral or enteral nutrition	28-day and 90-day all cause mortality	-Duration on ventilators -ICU stay -Infection incidence rate -Liver function and renal function -Diameter of midpoint of musculus rectus femoris -Serum concentration of albumin, pre-albumin, retinaldehyde binding protein, transferrin -Change of body composition	Nitrogen supply is as much as 2.5–3.0 g per kilogram (lean mass weight; EN/PN)	1.2–1.5 g per kilogram (lean mass weight; EN/PN)	80
02678325	PRCT	Switzerland	• Adult patients (age 18 years or older) • Expected stay at the ICU of 4 days upon admittance or longer • Expected enteral feeding during at least 4 days	-Amount of protein	-Total amount of calories -Nitrogen balance -Gastric residual -Number of diarrhea events -Occurence of constipation as measured in time without defecation	High protein enteral nutrition formula (caloric density of 1.2 kcal/ml and protein percentage 33% of the total caloric intake)	Standardized normal protein enteral nutrition formula (caloric density of 1.2 kcal/ml and protein 20% of the total caloric intake)	90
02865408	Open-label PRCT	Canada	• Mechanically ventilated adult patients (> 18 years old) admitted to ICU with an expected ICU dependency (alive and need for mechanical ventilation) • Vasopressor therapy, or mechanical circulatory support, at the point of screening of an additional 3 days, as estimated by the treating physician	Whole body protein balance	-Synthesis rates of hepatic secretory proteins -Biomarker of amino acid restriction or repletion -Metabolic substrates -Resting energy expenditure	1.75 g/kg/day of protein (enteral supplemented with IV amino acids)	1.0 g/kg/day of protein (enteral)	30
03021902	Phase II RCT	USA	Requiring mechanical ventilation with actual or expected total duration of mechanical ventilation ≥ 48 h Expected ICU stay ≥ 4 days after enrollment (to permit adequate exposure to the proposed intervention)	-Physical functioning	-Overall strength-upper and lower extremity -Quadriceps force-lower extremity strength -Hand held dynamometry -Distal strength-hand grip strength -Overall physical functional status -Mortality -Length of ventilation -ICU and hospital -ICU readmission -Re-intubation -Hospital-acquired infections -Discharge location (e.g., home vs rehab) -Body composition (ultrasound) -Health-related quality of life -Physical functioning (Katz Index of Independence in Activities of Daily Living) -Physical functioning (mental and cognitive functioning) -Health care resource utilization	IV amino acid (2.0–2.5 g/kg/day) + in-bed cycle ergometry	Usual care	142
03060668	Open-label PRCT	Brazil	•Critically ill patients Mechanically ventilated Expected length in the ICU > 3 days	Physical component of the SF-36	-Handgrip strength -ICU and hospital mortality	Caloric intakes determined by indirect calorimetry + 2.0–2.2 g/kg/day of protein	25 kcal/kg/day and 1.4 to 1.5 g/kg/day of protein	294
03160547	Multi-center pragmatic volunteer-driven registry-based randomized	Canada (over 100 international sites)	Nutritional high-risk Mechanical ventilation	60-day mortality	-Nutritional adequacy -Hospital mortality -Readmission to ICU and hospital -Duration of mechanical ventilation -ICU length of stay -Hospital length of stay	Higher prescription (≥ 2.2 g/kg/day) of protein (EN and/or PN)	A lower prescription (≤ 1.2 g/kg/day) of protein (EN and/or PN)	4000
03170401	PRCT	USA	Trauma/surgery Enteral nutrition expected ≥ 1 week	Serum transthyretin at 3 weeks after injury	-Ventilator-free days -Hospital-acquired pneumonia	Enteral protein supplementation	Standard enteral formula	500
03231540	PRC	Netherlands	• Admitted to intensive care • Mechanically ventilated • Expected duration of ventilation of 72 h • Expected to tolerate and require enteral nutrition for more than 72 h • SOFA score > 6 on admission day	In vitro loss of skeletal muscle function	-Loss of muscle function -Medical research council sum score -Changes in body composition (bioelectrical impedance analysis) -Loss of muscle mass (ultrasound of the quadriceps femoris muscle and diaphragm, questionnaires) -Quality of life	Whey protein supplement enriched enteral nutrition, with protein intake of 1.5 g/kg/day	Standard enteral nutrition, with protein intake of 1 g/kg/day	50
03319836	Retrospective	Canada	ICU patients	Daily total protein intake	-Caloric intake -Feeding interruptions ( tolerance) -Use of inotropes (pressors)	Very high protein enteral nutrition	Standard formula	40

Abbreviations: *PRCT* prospective randomized controlled trial, *ICU* intensive care unit, *MPR* mobility-based physical rehab, *EN* enteral nutrition, *PN* parenteral nutrition, *SOFA* Sequential Organ Failure Assessment

Meanwhile, owing to the potential risks of high amounts of proteins, the principle of precaution should prevail, i.e., the provision of 0.3–0.8 g proteins/kg/day during the early phase of critical illness. We definitely need to appraise more precisely the risk-to-benefit ratio by characterizing the relevant risks and measuring muscle function at the bedside as a proxy for the benefit of high protein intake.

## Abbreviations

EAT-ICU: Early Goal-directed Nutrition in ICU Patients
ICU: Intensive care unit
ICU-AW: ICU-acquired muscle weakness
PEPaNIC: Early versus late parenteral nutrition in the pediatric intensive care unit
